# Resolving multisensory conflict: a strategy for balancing the costs and benefits of audio-visual integration

**DOI:** 10.1098/rspb.2006.3578

**Published:** 2006-06-20

**Authors:** Neil W Roach, James Heron, Paul V McGraw

**Affiliations:** 1 Visual Neuroscience Group, School of Psychology, The University of NottinghamNottingham NG7 2RD, UK; 2 Department of Optometry, University of BradfordBradford BD7 1DP, UK

**Keywords:** multisensory integration, audio-visual conflict, Bayesian modelling

## Abstract

In order to maintain a coherent, unified percept of the external environment, the brain must continuously combine information encoded by our different sensory systems. Contemporary models suggest that multisensory integration produces a weighted average of sensory estimates, where the contribution of each system to the ultimate multisensory percept is governed by the relative reliability of the information it provides (maximum-likelihood estimation). In the present study, we investigate interactions between auditory and visual rate perception, where observers are required to make judgments in one modality while ignoring conflicting rate information presented in the other. We show a gradual transition between partial cue integration and complete cue segregation with increasing inter-modal discrepancy that is inconsistent with mandatory implementation of maximum-likelihood estimation. To explain these findings, we implement a simple Bayesian model of integration that is also able to predict observer performance with novel stimuli. The model assumes that the brain takes into account prior knowledge about the correspondence between auditory and visual rate signals, when determining the degree of integration to implement. This provides a strategy for balancing the benefits accrued by integrating sensory estimates arising from a common source, against the costs of conflating information relating to independent objects or events.

## 1. Introduction

Many physical properties of our external environment can be encoded by more than one sensory modality. Rather than being treated independently by the brain, it has long been recognized that these sources of information interact with one another. The perceptual consequences of these interactions are most noticeable when multisensory cues are placed in conflict. Classic demonstrations include marked shifts in the perceived location of auditory stimuli when accompanied by spatially distinct visual stimuli (the ‘ventriloquist illusion’, Pick *et al*. 1969; Welch & Warren 1980; Bertelson & Radeau 1981) and distortions of perceived visual rate induced by concurrent auditory stimulation (‘auditory driving’, Gebhard & Mowbray 1959; Shipley 1964; Myers *et al*. 1981; Welch *et al*. 1986; Recanzone 2003). Traditionally, the direction of such effects has been thought to reflect modality appropriate ‘capture’, with vision dominating spatial judgements and audition dominating temporal judgements. However, in recent years it has become clear that such a rigid strategy for resolving discrepancies between sensory estimates is unfeasible. Instead, it has been proposed that the brain may form an optimal combination of the available sensory information, based on the reliability of estimates derived from source.

Consider a situation in which an observer both hears and sees a sudden explosion. Though estimates of the spatial and temporal properties of the event derived by each modality are likely to be similar, each will be perturbed to some extent by sources of external (physical) and internal (neural) noise. Given this noisy input, the challenge for the observer is then to form a best approximation of what has occurred. Current opinion suggests that this is achieved via an integrative mechanism that operates according to maximum-likelihood estimation (MLE). According to a MLE model of multisensory integration, the strategy adopted by the brain is to merge sensory information into the most reliable composite estimate of a given property possible. If the noise associated with each sensory estimate is independent and normally distributed, the statistically optimum combination is a simple weighted average, where the degree to which each modality contributes to the ultimate multisensory percept is set according to the normalized reciprocal variance of the estimate it provides. For example, if the visual estimate of the location of the explosion is less variable (i.e. more reliable) than the corresponding auditory estimate, greater weight will be assigned to it during the integration process. However, if conditions such as smoke or haze from previous explosions degrade visual sensitivity to the extent that positional estimates become more variable (less reliable) than those provided by the auditory system, the pattern of weights will be reversed. In either case, the variance associated with the composite audio-visual estimate will be lower than for either of the individual sensory estimates. Thus, by exploiting the inherent redundancy of stimulus coding across sensory systems, this flexible strategy helps to minimize the effect that noise has on the observer's perceptual representations.

Empirical results consistent with near-optimal MLE integration of multisensory information have been reported in a number of studies (van Beers *et al*. 1999; Ernst & Banks 2002; van Beers *et al*. 2002; Gepshtein & Banks 2003; Alais & Burr 2004). In addition, comparable weighting schemes have been shown to predict observers' responses when presented with multiple visual cues to depth (Landy *et al*. 1995; Jacobs 1999; Rushton & Wann 1999), position (Landy & Kojima 2001) or surface geometry (Knill & Saunders 2003; Hillis *et al*. 2004), suggesting that similar processing strategies may operate for integrating information both within and across sensory modalities.

The advantages of MLE as a mechanism for multisensory integration are twofold. First, it provides a means of resolving discrepancies associated with internal and external noise, thus helping to maintain a unified percept of the world. Second, it has the capacity to increase the precision of perceptual representations, thereby facilitating the subsequent computation and execution of appropriate behavioural responses (Clarke & Yuille 1990; Ernst & Bülthoff 2004; Knill & Pouget 2004; Witton & Knudsen 2005). Critically, however, these benefits apply only when information relates to a common source. In rich, dynamic environments containing multiple stimuli, combining sensory information associated with independent objects or events is likely to be disadvantageous and in some instances hazardous. Thus, an inflexible stimulus-driven mechanism that automatically integrates multisensory information would carry potential costs as well as benefits.

Ideally, the brain would always be able to integrate sensory estimates derived from a common source, while avoiding the conflation of information derived from independent objects or events. Though not captured by a mandatory MLE model, there is reason to believe that there are strategies in place to maintain a balance between these competing goals. For instance, it has long been recognized that cross-modal interactions break down when the degree of conflict between each modality is large (Warren & Cleaves 1971; Jack & Thurlow 1973; Recanzone 2003; Bresciani *et al*. 2005; Gepshtein *et al*. 2005). Since highly discrepant sensory estimates are unlikely to relate to a common source, this acts to directly reduce the risk of integrating unrelated information. In addition, there is evidence to suggest that even when integration does take place, the brain does not necessarily discard unimodal information altogether. Indeed, based on results from a task in which observers were asked to discriminate between visual–haptic stimuli using any means available, Hillis *et al*. (2002) suggest that *either* the combined estimate or one of the unimodal estimates can be accessed, depending on which is most advantageous for a given judgement.

In this study, we investigated interactions between auditory and visual temporal rate perception while instructing observers to base their judgements solely on information from one modality. This approach differs from most studies investigating MLE integration, where observers are invariably asked to make single judgements about discrepant multisensory stimuli. Interestingly, under these conditions we find that the magnitude of cross-modal effects are neither consistent with mandatory MLE integration nor with uncompromised access to the relevant unimodal estimate. Rather, observers' rate percepts fall between the predictions of each strategy, suggesting that only partial integration of temporal information is occurring. Additionally, a key advantage of this approach is the facility to map out audio-visual interactions over a wide range of inter-modal discrepancies, revealing a gradual transition between partial cue integration and complete segregation. Building upon recent suggestions (Ersnt 2005), we go on to develop a simple Bayesian model of audio-visual integration that accounts for these new findings. Furthermore, we show that this parsimonious computational approach can be used to predict observer performance under novel stimulus conditions.

## 2. Methods and results

### (a) Observers

Two of the authors (NWR and JH) acted as observers along with one participant (EGL) who was completely naive to the purposes of the experiment. Each had normal or corrected to normal vision and no hearing loss.

### (b) Stimuli

Visual stimulation was produced using a 14 mm diameter green light-emitting diode (LED), positioned 1 m in front of the observer. The LED had a maximum luminance of 6400 cdm^−2^ and flickered on and off at a controllable rate. Auditory stimuli were bursts of white noise sampled at 8192 Hz and presented binaurally via Sennheisser HD-265 headphones. To produce a comparable temporal profile to the flickering visual stimulus, each noise burst was amplitude modulated by a square wave around a fixed mean intensity (65 dB SPL). Auditory stimuli were produced at a variety of modulation depths, expressed here as a multiple of each observer's detection threshold (initially obtained by measuring the minimum depth which could be distinguished from a non-modulating stimulus with 75% accuracy).

### (c) Unimodal rate discrimination

**Figure 1:**
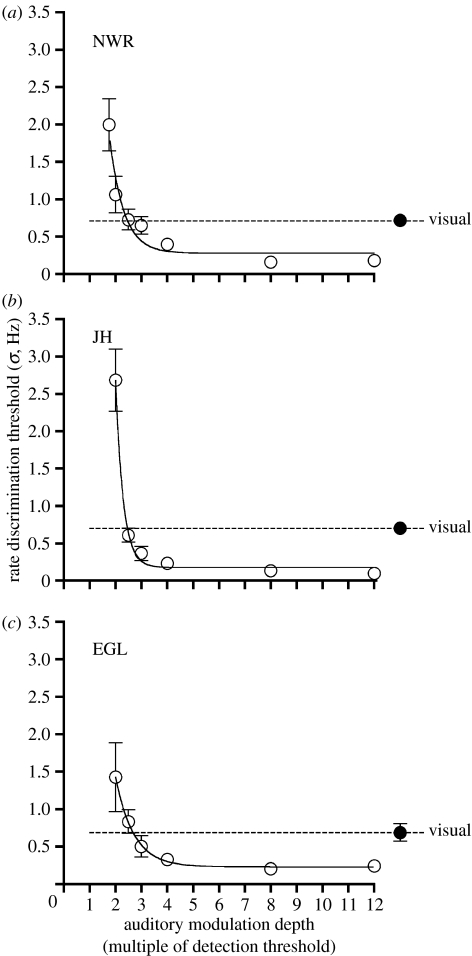
Thresholds for discriminating the rate of modulation of auditory (unfilled symbols) and visual (filled symbols) stimuli relative to a 10 Hz standard. Auditory thresholds are shown as a function of modulations depth, expressed as a multiple of each observer's detection threshold. Error bars in this and subsequent figures indicate ±1 standard error, estimated by a bootstrap procedure. Note, no systematic biases were observed in any condition.

Ability to discriminate the rate of visual or auditory modulation was first measured relative to a fixed 10 Hz standard. A two-interval forced choice procedure was employed, whereby observers judged which of two successive one-second intervals contained the stimulus with the faster rate. The order of presentation of test and standard intervals was randomized on a trial-by-trial basis. A method of constant stimuli was employed (seven test rates centred on 10 Hz, 40 trials per test rate) and psychometric functions were modelled by fitting a cumulative Gaussian function to each of the resulting datasets. Separate runs measured discriminative ability for visual modulation and for each of a number of auditory modulation depths. As shown in figure 1, auditory rate discrimination thresholds varied systematically as a function of modulation depth. With larger modulation depth stimuli, auditory rate judgements were more precise than visual judgements. However, by reducing modulation depth auditory thresholds could be made to approximate or exceed visual thresholds for each individual observer. Accordingly, manipulation of the modulation depth of the auditory stimulus provided a means of controlling the relative balance between visual and auditory sensitivity.

### (d) Cross-modal interactions with equated auditory and visual sensitivity

In cross-modal conditions, observers were required to discriminate rate information derived from one modality (task-relevant), while ignoring rate information presented to the other (task-irrelevant). In each case, judgements were made relative to a congruent bimodal reference stimulus comprising visual and auditory modulation at 10 Hz. The auditory and visual components of the reference stimulus were presented in phase, such that periods in which the LED was on were temporally coincident with periods in which the auditory stimulus was loudest. Psychometric functions were obtained for a range of interleaved task-irrelevant test rates using identical procedures to those used in unimodal measurements. Changes in perceived rate induced by task-irrelevant stimuli were quantified by measuring shifts in the point of subjective equality (PSE), the physical test rate required in the task-relevant modality to be perceptually equivalent to the standard.

**Figure 2:**
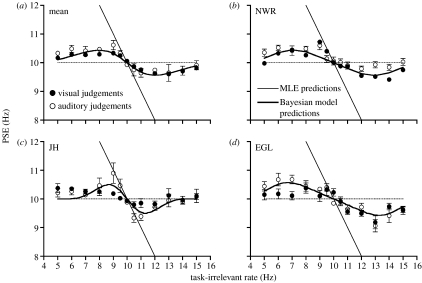
Interactions between visual and auditory rate perception under conditions of equated unimodal sensitivity. Observers were required to make judgements based on either auditory (unfilled symbols) or visual (filled symbols) information, while ignoring task-irrelevant stimuli presented to the other modality. Data points indicate the physical rate in the task-relevant modality required to be perceptually equivalent to a 10 Hz standard. The solid oblique line in each panel indicates the predicted results based on maximum-likelihood estimation. Solid curves show predictions of the best-fitting Bayesian model for each observer (see main text for details).

Cross-modal data was first collected under conditions where auditory and visual sensitivity was equated. The modulation depth of auditory stimuli was set to the point at which the exponential fit of the auditory threshold data in figure 1 intersects the dotted horizontal line indicating visual threshold level for each observer. Figure 2 displays results for visual judgements (filled symbols) and auditory judgements (unfilled symbols) and shows that PSEs were systematically pulled above and below the reference frequency, depending on the rate of the task-irrelevant stimulus. For instance, in order for perceived visual rate to be equivalent to the reference stimulus, physical visual flicker rates *greater* than 10 Hz were required when paired with slow irrelevant auditory stimuli and flicker rates *less* than 10 Hz were required when paired with fast auditory stimuli.

The fact that PSEs were systematically altered by an irrelevant stimulus strongly suggests that observers were not able to retain uncompromised access to the individual auditory and visual rate estimates. Rather, some form of integration of rate information has occurred. However, the magnitudes of the shifts in perceived rate are not consistent with mandatory implementation of MLE. Since auditory and visual sensitivity were equated, MLE would predict equivalent weighting of information from each modality (i.e. a simple arithmetic average). The resulting linear prediction is shown in figure 2 across a range of task-irrelevant rates spanning 2 Hz either side of the 10 Hz reference. MLE over-estimates the amount of shift in the PSE away from the reference rate.

While mandatory MLE would predict that the degree of cross-modal distortion in both judgement conditions should continue to rise as the irrelevant stimulus rate is moved away from the 10 Hz reference, this is not borne out in the data (see figure 2). In contrast, effects in both conditions display a finite tolerance to bimodal rate discrepancies. Minimal changes in perceived rate were induced by task-irrelevant rates that were considerably slower (i.e. 5 Hz) or faster (i.e. 15 Hz) than the reference stimulus.

**Figure 3:**
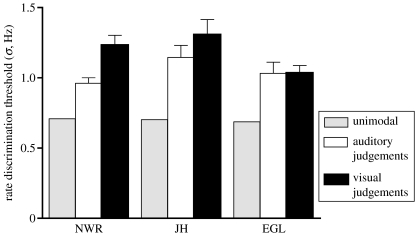
Comparison of auditory and visual rate discrimination thresholds with and without task-irrelevant stimuli presented to the other modality. Mean thresholds calculated across task-irrelevant rate conditions are shown for each observer (±1 standard error).

Since the rates of auditory and visual stimuli in each test interval were uncorrelated, cross-modal interactions came at an overall cost to the accuracy of rate judgements. As shown in figure 3, rate discrimination thresholds for both auditory and visual judgements exceeded those obtained under unimodal conditions.

### (e) A simple Bayesian model

The results of our cross-modal experiment indicate that in the majority of conditions, auditory and visual rate information was neither merged into a composite rate estimate nor processed in complete independence of its counterpart in the other modality. To account for these results, here we implement a Bayesian model of multisensory integration that incorporates uncertainty about both the relationship between unimodal rate estimates as well as the estimates themselves. We assume that observers combine information derived from the noisy auditory (*A*) and visual (*V*) representations with prior knowledge that has been built up about the co-occurrence of particular combinations of auditory (*a*) and visual (*v*) rates to infer the most likely physical stimulus. The *posterior* distribution *P*(*a*,*v*|*A*,*V*) specifies the probability of perceiving rates *a* and *v* given the noisy estimates *A* and *V*. According to Bayes' rule,

2.1 $$P(a,v|A,V)=\frac{1}{\alpha_{1}}P(A,V|a,v)P(a,v),$$

where *P*(*A*,*V*|*a*,*v*) indicates the *likelihood* that particular auditory and visual representations will result from a given physical stimulus; *P*(*a*,*v*) specifies *prior* knowledge about the probable correspondence between auditory and visual rates and *α*_1_ is a normalization constant that ensures that the posterior probability distribution sums to 1. Assuming a least squares *loss* function, one can calculate the optimal auditory and visual percepts as the centroid of the two-dimensional posterior distribution.

In keeping with previous models, we assume that the noise associated with each sensory estimate is independent and normally distributed. Accordingly, likelihood distributions can be derived from observers' unimodal rate discrimination thresholds (*σ*_*A*_ and *σ*_*V*_) as follows:

2.2 $$P(A,V|a,v)=\frac{1}{\alpha_{2}}{\text{e}}^{-\frac{1}{2}\left(\frac{{\left(A-a\right)}^{2}}{{\sigma_{A}}^{2}}+\frac{{\left(V-v\right)}^{2}}{{\sigma_{V}}^{2}}\right)}.$$

In everyday life, concurrent audio-visual signals often, but not always, relate to a common source. While mandatory MLE integration assumes perfect correspondence between auditory and visual rates (*a*=*v*), here we incorporate a more flexible prior that reflects this variability. Specifically, we model the prior probability distribution as the amalgamation of two components: a ‘linked’ component consisting of a Gaussian function of the difference between auditory and visual rates, centred on precise correspondence, and an ‘independent’ component comprising of a uniform distribution across combinations of rates in each modality,

2.3 $$P(a,v)=\frac{1}{\alpha_{3}}\left(\omega +{\text{e}}^{-\frac{{\left(a-v\right)}^{2}}{2{\sigma_{av}}^{2}}}\right).$$

The parameter *σ*_*av*_ controls the spread of the Gaussian component around the identity line, while the parameter *ω* sets the probability level of the uniform component distribution relative to the peak of the Gaussian. Notionally, the prior represents accumulated knowledge about the relationship between auditory and visual rate signals built up through repeated exposure to both correlated and uncorrelated sources in the world.

Using the model, predicted outcomes for the cross-modal task were generated. To estimate the prior distribution for each observer, we calculated the values of *ω* and *σ*_*av*_ that produced the best-fitting (least-squares residual) predictions of the combined visual and auditory judgement datasets. As shown by the solid curves in figure 2, these predictions do a far superior job to MLE in capturing both the overall magnitude of the observed interaction effects and the limited tolerance shown to inter-modal discrepancies.

**Figure 4:**
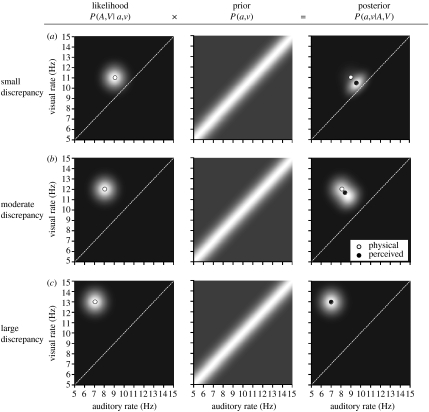
Schematic showing the operation of the Bayesian model under different levels of inter-modal discrepancy. Probability values in each two dimensional distribution are represented by varying grey-scale levels, with lighter regions indicating higher-probability values. For reference purposes, the dotted diagonal lines indicate points of equivalent auditory and visual rate. Noisy stimulus information, represented in the likelihood distribution (left-hand column) is combined with prior assumptions about the correspondence between auditory and visual rate signals (central column) to generate a posterior distribution (right-hand column) of potential percepts. Physical and optimal perceived rates in each modality are indicated by the positions of the small unfilled and filled circles, respectively. (*a*) Substantial distortions of perceived rate are predicted when the degree of discrepancy between auditory and visual rates is small. However, as the degree of discrepancy is increased, the amount of distortion will (*b*) decrease and (*c*) ultimately disappear.

To illustrate the main components and operation of the model, a graphical representation is shown in figure 4. In each panel, lighter regions designate higher probability values than darker regions. Row (*a*) shows a hypothetical situation in which a 9 Hz auditory stimulus is paired with an 11 Hz visual stimulus. The combination of physical rates dictates the centre of the likelihood function, as indicated by the position of the small-unfilled circle. Perceived auditory and visual rates are calculated by taking the centroid of the posterior distribution, indicated by the position of the small black circle. In the case of complete integration of auditory and visual information, auditory and visual percepts would be fused such that this estimate would fall upon the dashed diagonal identity line. However, since our prior does not assume perfect correspondence between rate information in the two modalities, the predicted perceptual experience falls in between independence and complete integration.

As long as combinations of auditory and visual stimuli fall near the identity line, the posterior distribution is dominated by the Gaussian linked component of the prior, producing distortions of perceived rate that increase with the degree of discrepancy between modalities. However, as shown in row (*b*), posterior functions for discrepant stimuli falling towards the limits of the linked prior become increasingly affected by the independent component, resulting in smaller effects. Further increase in the degree of discrepancy between auditory and visual rates will ultimately negate the influence of the linked prior component entirely. Row (*c*) demonstrates that under these circumstances, the model predicts veridical rate perception in both modalities.

A couple of points warrant mention here. First, it is important to note that without the uniform component of the prior, the model would fail to predict the tuning of interaction effects as a function of rate discrepancy. If one were to implement the linked (Gaussian) component of the prior in isolation, the model would produce partial integration of rate estimates. However, as with mandatory MLE, the magnitude of predicted interaction effects would remain a linear function of rate discrepancy and fail to capture the observed tolerance profiles. Second, successful prediction of the experimental data would not be possible if rate percepts were derived from a maximum *a posteriori* estimate. Because of the composite nature of the prior, posterior probability distributions formed by the model are sometimes bimodal. This presents two problems: (i) the predicted transition between partial integration and segregation seen with increasing discrepancy becomes abrupt, rather than gradual and (ii) in some conditions it becomes impossible to find any combination of rates which will give rise to a perceived rate of 10 Hz in the task-relevant modality.

### (f) Cross-modal interactions with unbalanced auditory and visual sensitivity

Having established estimates of the prior distributions for each observer, we next sought to determine whether the Bayesian model could predict performance under new stimulus conditions. To do this, we repeated the cross-modal experiment while manipulating the precision of auditory rate estimates relative to those formed by the visual system. As with all Bayesian approaches, the model dictates that perception is a trade-off between the reliability of a particular estimate (represented by the likelihood) and the prior. Reducing the precision of auditory rate estimates should flatten the likelihood along the auditory dimension, making perception more susceptible to influence by the prior. As a result, the model predicts that greater distortion of auditory rate judgements by visual stimuli should occur. Increasing the estimate precision should have the opposite effect, resulting in percepts that are less prior driven (i.e. more veridical).

**Figure 5:**
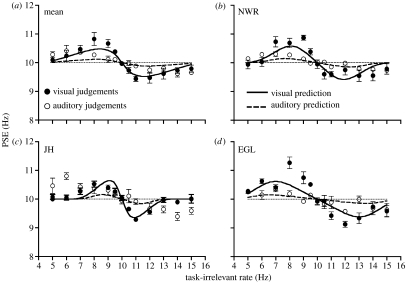
Interactions between visual and auditory rate perception under conditions of superior auditory sensitivity. Smaller distortions of perceived rate are evident for auditory judgements (unfilled symbols), than for visual judgements (filled symbols). Curved functions show the predictions of the Bayesian model. Each prediction is based on unimodal sensitivity data and prior estimates derived from the initial cross-modal experiment and contains no free parameters.

**Figure 6:**
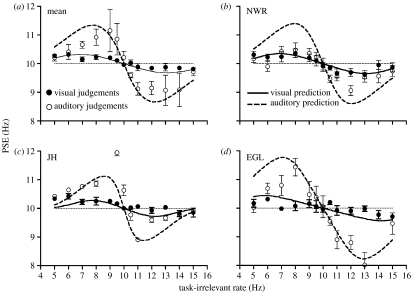
Interactions between visual and auditory rate perception under conditions of inferior auditory sensitivity. Larger distortions of perceived rate are evident for auditory judgements (unfilled symbols), than for visual judgements (filled symbols). Curved functions show the predictions of the Bayesian model.

From the exponential curve fits of auditory unimodal data shown in figure 1, the modulation depth of auditory stimuli were set such that auditory rate discrimination thresholds were either 50 or 200% of visual thresholds for each observer. Cross-modal interactions were then independently measured for each stimulus set, using identical methods to those described previously. For each observer, model predictions were also generated using the prior parameters obtained in the previous experiment, along with the new set of unimodal rate discrimination thresholds. Experimental data and model predictions for the ‘higher-auditory precision’ (*σ_A_*=0.5*σ_V_*) and ‘lower auditory precision’ (*σ_A_*=2*σ_V_*) conditions are shown in figures 5 and 6, respectively. While PSE functions retain the same characteristic shape seen in the previous cross-modal experiment, clear differences are now apparent between the magnitude of cross-modal effects in auditory and visual judgement conditions. When auditory precision was increased (figure 5), the distortion of perceived rate was smaller for auditory judgements than visual judgements. Reducing auditory precision (figure 6) had the opposite effect, resulting in larger distortions of perceived auditory rate than visual rate. These changes in the relative magnitude of the interaction effects were correctly predicted by the model, which produced plausible approximations of the mean datasets in each case. Some departures from model predictions can be seen in the individual datasets (most noticeably for JH in figure 5 and NWR in figure 6). However, these discrepancies are not systematic across observers and it should be stressed that the predicted functions involve no free parameters and are thus not a ‘fit’ of the data.

## 3. Discussion

The experiments reported here add to a large body of literature documenting cross-modal interactions between visual and auditory temporal perception. Previous studies have invariably found that such effects are unidirectional: perceived visual timing is found to be pulled towards that of a discrepant auditory stimulus whereas perceived auditory timing remains unaffected by discrepant visual stimuli (Gebhard & Mowbray 1959; Shipley 1964; Myers *et al*. 1981; Welch *et al*. 1986; Shams *et al*. 2002; Recanzone 2003). In contrast, here we show that by matching the relative sensitivity of the two modalities, distortions of perceived auditory rate can be induced which are equivalent to those seen for visual judgements. To our knowledge, this is the first demonstration that cross-modal interactions between auditory and visual rate perception can occur in both directions.

Similar results have recently been reported in the spatial domain, where it has been shown that the visual dominance over positional judgements can be attenuated (Battaglia *et al*. 2003) or even reversed (Alais & Burr 2004) by degrading visual sensitivity. There are, however, critical differences between these previous findings and those in the present study. Alais & Burr report that when asked to make single positional judgements about slightly discrepant audio-visual stimuli, observers respond in accordance with near optimal MLE. That is, observers appear to form an average of the two positional estimates after weighting each according to its reliability. However, in the present study we demonstrate that a comparable averaging mechanism cannot account for results obtained when observers make separate auditory and visual rate judgements. Under these conditions we find that the magnitude of cross-modal interactions is considerably smaller than would be predicted by mandatory implementation of MLE. Since this difference holds for both auditory and visual judgements, our results also differ from the alternative model proposed by Battaglia and colleagues in which reliability-based weighting is supplemented by a predisposition towards one modality. Rather, to reconcile our results with a simple averaging mechanism, one would need to assume that weights could be flexibly altered so as to bias integration towards which ever modality is relevant to the task at hand. Alternatively, it could be suggested that a representation-switching strategy is being implemented, whereby observers alternate between using a unimodal rate estimate on some trials and a combined auditory-visual MLE estimate on others. While there is not currently sufficient evidence to discount these possibilities entirely, a more cogent explanation of the present results is that auditory and visual rate information are only *partially* integrated. In support, some evidence for partial integration has recently been reported for a categorical audio-visual task (Shams *et al*. 2005).

A further limitation of mandatory MLE as a model of multisensory processing is that it fails to account for the fact that cross-modal interactions often break down when information provided by each modality is highly conflicting (Warren & Cleaves 1971; Jack & Thurlow 1973; Recanzone 2003; Bresciani *et al*. 2005). In previous experiments where observers have been asked to make single combined judgements about multisensory stimuli, researchers have typically avoided this issue by introducing only small, undetectable discrepancies along the dimension of interest. Since our experimental design did not force observers to combine auditory and visual information, we were able to measure interaction effects across a wide range of discrepancies. This revealed tolerance profiles characterized by a gradual transition towards segregation of sensory information with increasing discrepancy. Our data suggest that tolerance profiles are relatively invariant to changes in the type of judgement and the balance between relative unimodal sensitivity.

Following suggestions made by Ernst (2005), we implement a Bayesian model that infers perceived rate by combining noisy sensory estimates with prior knowledge about the correspondence between signals in each modality. In contrast to mandatory MLE, this approach does not presuppose obligatory integration of multisensory information. Instead, perceptual experience may fall anywhere along a continuum ranging from complete segregation of sensory estimates to complete integration. By assuming a prior whereby auditory and visual rates are often (but not always) equivalent, the model successfully captured patterns of partial integration of auditory and visual rate information across a wide range of inter-modal discrepancies, as well as for novel stimulus conditions.

Knowledge of the probable occurrence of different combinations of auditory and visual rates is unlikely to be innate, but, rather, built up through extended experience with the world (Adams *et al*. 2004). Co-occurring auditory and visual temporal signals are often similar, as they commonly relate to the same external object or event. However, from time to time, uncorrelated signals will co-occur by chance, where each emanates from an independent source. The Bayesian approach dictates that degree of multisensory integration will be set in direct proportion to the strength of correspondence between sensory signals. High degrees of correspondence will produce tightly tuned prior distributions and, consequently, result in significant integration. In contrast, infrequent co-occurrence between signals will result in a broadly tuned prior distribution and little or no integration. This provides a practical strategy for striking a balance between deriving benefit from the integration of estimates derived from a common source, while avoiding the costs of integrating estimates derived from independent sources.

In addition to setting the degree of integration between sensory estimates, prior knowledge about the correspondence between sensory signals in the Bayesian model also determines the degree of tolerance shown to inter-sensory discrepancies. As would be expected given a constant prior for audio-visual rate, tolerance profiles shown in the present study displayed little variation across a range of stimulus conditions. However, different prior distributions would be needed to reflect the correspondence between other stimulus properties (e.g. position) or other sensory modalities (e.g. visual–haptic), resulting in independent predictions about tolerance profiles. The model predicts that strong patterns of integration induced by tight correspondence between sensory signals should be accompanied by low tolerance towards sensory discrepancies. In contrast, poor correspondence will produce weaker integration over a wider range of discrepancies. Future empirical studies testing these predictions will ultimately inform us as to the veracity of this approach.

In the present study, we have focused purely on discrepancies between auditory and visual signals along the judgement dimension (i.e. temporal rate). However, it is quite possible that the degree of integration between rate estimates might also depend on other factors, such as the spatial proximity of the two sources. Since our auditory stimuli were presented diotically (same signal in each ear), the perceived location of each sound was centred on the observers' midline, comparable with the position of the visual LED. However, headphone presentation necessitates that sounds are perceived intracranially (located within the head). Although this lack of externalization does introduce a form of spatial discrepancy between visual and auditory stimuli, in pilot experiments we found that patterns of interactions effects were comparable to when auditory stimuli were presented via an external speaker mounted to the LED.

How might a Bayesian model of multisensory integration be implemented at a neural level? Traditionally, multisensory integration has been viewed as a feed-forward process, whereby projections from sensory-specific neural regions converge upon multimodal sites. Within this framework, it is difficult to reconcile how different sensory estimates could influence one another, yet still remain as separate entities. However, neuroimaging and physiological studies have begun to undermine the plausibility of a purely feed-forward system, by demonstrating that changes in cortical activity within traditional unimodal areas can be induced by inputs to other sensory systems (Calvert *et al*. 1997; Macaluso *et al*. 2000; Schroeder *et al*. 2001; Fu *et al*. 2003). Modulation of unimodal signals could be mediated by feedback projections from multimodal regions (Driver & Spence 2000; Meredith 2002), or alternatively, by direct interconnections between primary sensory areas (Falchier *et al*. 2002; Rockland & Ojima 2003). In either case, these changes in unimodal processing could provide a feasible mechanism through which partial integration of sensory information might occur. Central to all Bayesian models is the probabilistic representation of sensory information and prior knowledge. While a number of suggestions have been made as to how these distributions might be implemented at a neural level (e.g. the rate of spiking, or its variability, across neural populations; see Knill & Pouget 2004; Witten & Knudsen 2005 for recent reviews), the precise mechanisms remain unknown and their elucidation represents a major challenge in this field.
